# Supplementary figures and images for: Visual System Hyperexcitability and Compromised V1 Receptive Field Properties in Early-Stage Retinitis Pigmentosa in Mice
Source: eNeuro. 2022 Jun 23;9(3):ENEURO.0107-22.2022. doi: 10.1523/ENEURO.0107-22.2022 (PMC9239850; doi:10.1523/ENEURO.0107-22.2022)

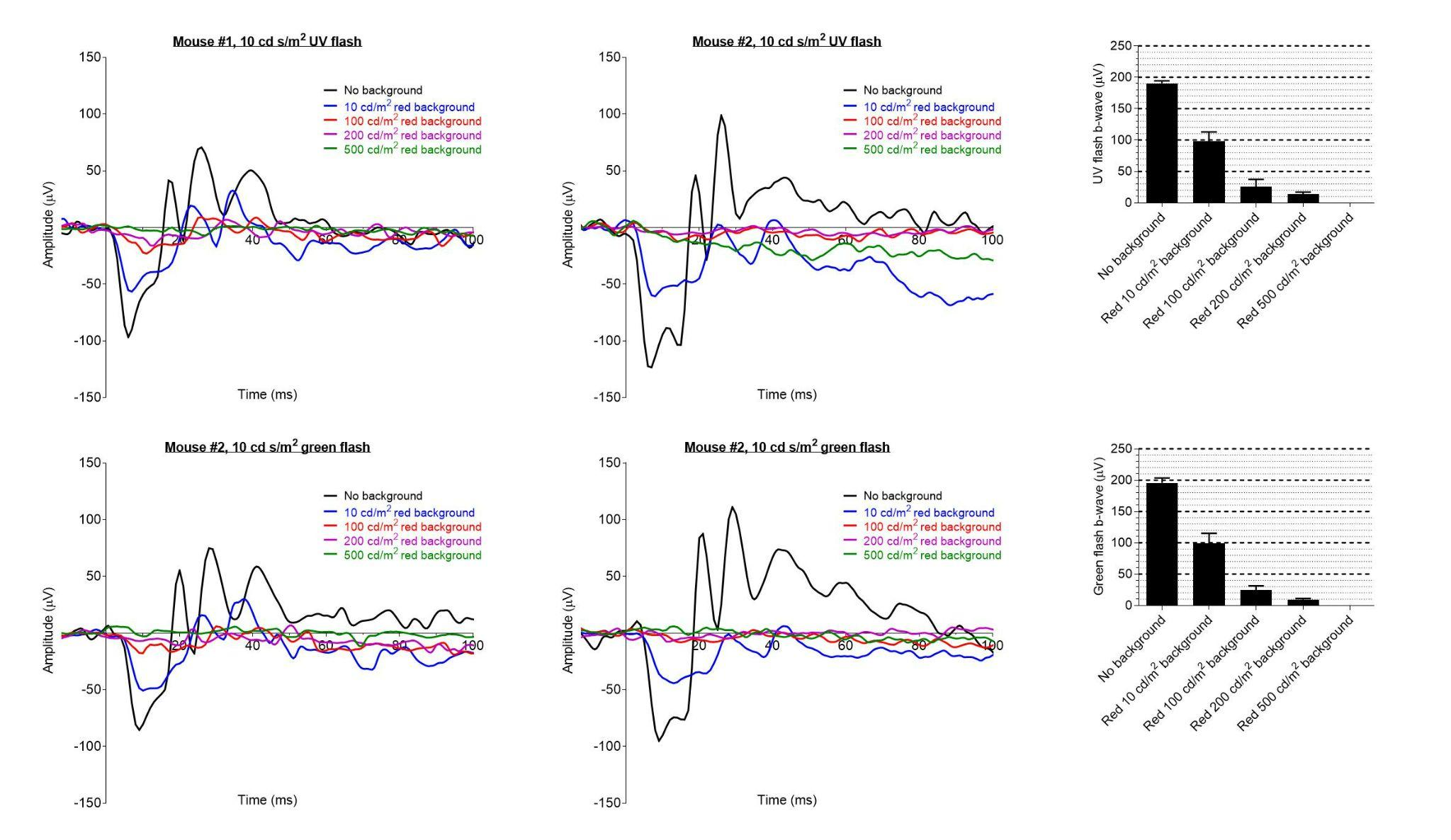

Supplement: Extended Data Figure 1-1 — Steady red illumination suppresses rod-driven ERG in cone-transducin knock-out (Gnat2–/–) mice. Gnat2–/– mice were dark-adapted overnight before the recording. Mice were first recorded in a dark-adapted state (no background), and then steady red light at 10, 100, 200, and 500 cd/m2 was introduced, and ERGs were repeated in an increasing “red light-adapted” state. Each step had an adaptation period of 60 s. Two stimuli were presented for each step using an interstimulus interval of 90 s. Note that the ERG response in 200 cd/m2 background illumination is less than 10% from the dark-adapted state, and the response in 500 cd/m2 background illumination cannot be distinguished from the noise. Download Figure 1-1, TIF file. [file enu-eN-NWR-0107-22-s01.tif]
